# Use of balloon tamponade in management of vaginal laceration and its possible complication of urinary stress incontinence: a case report

**DOI:** 10.1186/s12884-020-02885-0

**Published:** 2020-04-15

**Authors:** Choi Wah Kong, William Wing Kee To

**Affiliations:** Department of Obstetrics and Gynaecology, United Christian Hospital, 130 Hip Wo Street, Kwun Tong, Hong Kong SAR

**Keywords:** Lacerations, Postpartum hemorrhage, Balloon tamponade, Urinary incontinence, Case report

## Abstract

**Background:**

Postpartum haemorrhage from vaginal lacerations can occasionally be refractory to suturing and vaginal packing. Bakri uterine balloon has been widely adopted to stop uterine bleeding, but its use to stop bleeding in vaginal lacerations and its possible complications have seldom been reported.

**Case presentation:**

We report a patient who had vacuum delivery for fetal distress and subsequently had postpartum hemorrhage due to previous caesarean uterine scar rupture and multiple vaginal lacerations. The severe bleeding persisted despite total abdominal hysterectomy, pelvic embolization and vaginal gauze packing, but was finally controlled by a Bakri balloon tamponade inserted into the vagina. The patient suffered from severe stress incontinence after delivery. The possible use of balloon tamponade in vaginal lacerations and the different types of vaginal balloons that are available in the market for this purpose are reviewed. The possible causes leading to stress incontinence is reported to alert the obstetrician that such management is not free of complications.

**Conclusion:**

The use of Bakri balloon can help to control bleeding in severe vaginal lacerations that are unresponsive to traditional vaginal gauze packing. Further studies are needed to evaluate the risks of stress incontinence as a possible complication of vaginal balloon tamponade.

## Background

Vaginal lacerations can cause severe postpartum hemorrhage (PPH) after vaginal delivery particularly after instrumental deliveries. Haemostatic stitches are difficult to apply when there are multiple lacerations located high up in vaginal fornices, or are ineffective when vaginal mucosa is friable and oedematous. Bakri uterine balloon has been widely adopted to stop uterine bleeding in postpartum hemorrhage, but there are few reports available that showed its effectiveness to stop bleeding in vaginal laceration.

We report a patient who had PPH after vacuum delivery due to previous caesarean scar rupture and vaginal lacerations. The bleeding from vaginal lacerations was finally stopped by Bakri balloon, but patient had severe stress incontinence afterwards. To our knowledge, this is the first case report of using Bakri balloon for vaginal lacerations which was complicated by stress incontinence afterwards.

## Case presentation

A 30- year-old para 1 woman, had an uncomplicated lower segment caesarean section in her first pregnancy for fetal distress. The baby weighed 3.1 kg. The antenatal course was uneventful in this second pregnancy and she opted for a trial of vaginal delivery. She did not have any urinary incontinence during pregnancy. She was admitted to hospital at 41 week of gestation for postdate pregnancy. Her cervix was favourable, artificial rupture of membranes was performed and oxytocin infusion was commenced for induction of labour. After a first stage of 5.5 h, the cervix was fully dilated but prolonged fetal bradycardia down to 80 bpm was noted in the cardiotocogram during active pushing. Vacuum extraction was performed and a baby weighing 3.09 kg was born in good condition, with an Apgar score of 10 at 1 and 5 min of birth. Cord arterial pH was 7.19 with base excess − 7.6 mmol/L. The episiotomy wound and a deep right vaginal laceration were repaired. However, she had PPH with hypovolemic shock shortly afterwards. Fluid resuscitation and syntocinon infusion were given, and exploration of the uterus found no retained placental tissues. Intramuscular carboprost was given and haemostatic stitches were applied to the vaginal wound. A Bakri balloon was inserted into the uterine cavity with 300 ml saline infused. However, patient remained haemodynamically unstable and haemogloblin level dropping from 11.7 to 7.1 g/ dL despite blood transfusion. There was persistent drainage from the uterine balloon signifying uncontrolled uterine atony, with estimated blood loss since delivery around 2400 ml. The patient soon developed disseminated intravascular coagulopathy. The platelet count decreased to 133 × 10^9/L and the fibrinogen level decreased to 1.6 g/L. The prothrombin time was prolonged to 16.4 s while the activated partial thromboplastin time was prolonged to 52.3 s. Platelet and fresh frozen plasma were transfused. Laparotomy was performed, and a 2 cm defect at the left side of the previous caesarean scar with a haematoma beneath the vesico-uterine fold was found, compatible with rupture of the uterine scar. The patient was in hypovolemic shock with active bleeding from the rupture site and the uterine atony was not responsive to medical treatment. Total abdominal hysterectomy was performed and multiple haemostatic stitches were applied to the vaginal wound. One roll gauze soaked with adrenaline was packed into the vagina. The operative blood loss was 3600 ml. The patient was transferred to intensive care unit after operation.

However, the patient had continued bleeding from the pelvic drain and from the vagina after operation. Further transfusion of blood products was given and pelvic embolization was performed 7 h after the hysterectomy. Bilateral internal iliac arteries were canalized. The left internal iliac branches were apparently intact, but extravasation of contrast from two right uterine branches arising from the inferior gluteal artery of the anterior trunk of the right internal iliac artery was noted and embolized. However, continued bleeding from the vaginal lacerations persisted with persistent disseminated intravascular coagulopathy, and re-laparotomy was performed 20 h after the hysterectomy. Abdominally, there was a 6 cm haematoma in the bladder peritoneum with active bleeders over the anterior vaginal vault. Multiple haemostatic stitches, Floseal followed by manual compression, gauze packing with Monsel’s solution, and a Cook’s cervical ripening balloon infused with 100 ml saline inserted into the vagina all failed to control the bleeding. A Bakri’s uterine balloon was inserted into the vagina with 300 ml saline infused. One stitch was applied to oppose the bilateral vulva to prevent the Bakri balloon from slipping out. Additional haemostatic stiches were applied in the vault abdominally and Tachosil was applied to the bleeding site. The pelvic cavity was packed with 3 pieces of abdominal gauze packs before the abdominal wall was closed. The operative blood loss was 5000 ml. The patient subsequently remained haemodynamically stable with no further bleeding. Re-laparotomy was performed 30 h afterwards and the pelvic packing and vaginal Bakri balloon were removed without further bleeding. The patient had transient fever in the postoperative period but responded to intravenous antibiotics.

The patient noticed severe stress and urge incontinence on day 9 after delivery after removal of the foley catheter, necessitating the use of incontinence napkins throughout the day. Investigations showed no urinary tract infection and no ureteric nor bladder injury. The patient was taught to perform pelvic floor exercise and was discharged on day 25 with mild improvement in the incontinence symptoms. Further partial improvement in her incontinence was observed in the following 3 months. Urodynamic studies found normal bladder capacity and sensation, with mild elevation of detrusor pressure at the latter part of the filling phase, and stress incontinence was demonstrated. She continued the pelvic floor exercise and her symptoms further improved gradually. At 10 months after delivery, she had only mild stress incontinence when coughing and jumping with no urge symptoms. She was satisfied with her condition and declined further surgical treatment.

## Discussion and conclusions

Uterine atony and vaginal lacerations can jointly cause severe PPH after vaginal delivery particularly after instrumental deliveries. In our patient, despite total abdominal hysterectomy to control the haemorrhage from uterine atony and caesarean scar rupture, pelvic embolization and vaginal gauze packing still failed to stop the bleeding from the vaginal lacerations, particularly when she developed disseminated intravascular coagulopathy as a complication of massive haemorrhage.

Foley catheters have commonly been used to stop bleeding from vaginal lesions in the past, but there are no formal case reports documenting such successful attempts in the literature. The use of balloon tamponade to control bleeding in vaginal lacerations was firstly documented by Condie et al. in 1994 using a Sengstaken tube in a 14-year-old girl who had multiple vaginal lacerations after sexual intercourse when the bleeding was uncontrolled by suturing, vaginal packing and bilateral internal artery ligation [1]. Pinborg et al. first described the successful use of vaginal tamponade in obstetric patients with the use of a blood pressure cuff [2]. Two patients had vaginal haematoma after normal vaginal delivery, but severe bleeding recurred despite drainage of the haematoma and direct manual compression. A blood pressure cuff wrapped by a sterile glove was packed into the vagina, which was then inflated to 120 mmHg to stop the bleeding. Similar successful use of blood pressure cuff was also reported by Cameron et al. in a patient who had vaginal lacerations after forceps delivery [3].

Bakri balloon (Cook Medical, USA) is a silicon uterine specific inflatable balloon tamponade system designed to be inserted into the uterine cavity [4], and has been shown to be highly effective in management of PPH due to uterine atony or placenta praevia to reduce the need for peripartum hysterectomies [5]. While the Bakri balloon was widely used as the first uteine-sparing surgical treatment of PPH after failed medical treatment by uteronic drugs, there are only two case reports in the literature describing its use in vaginal lacerations. Tattersall et al. described a patient with vaginal haematoma and several large vaginal lacerations after normal vaginal delivery [6]. Two Bakri balloons were placed in the vagina with the superior and inferior balloons inflated with 400 and 350 ml saline respectively. Two sutures were placed in the vulva to prevent balloon expulsion. The Bakri balloons were removed 30 h after insertion and no more bleeding was noted. Yoong et al. described placing a Bakri balloon in a patient with several vaginal tears after vacuum delivery with 100 ml saline infused into the balloon, and vaginal gauze was packed distal distal to the balloon to prevent balloon expulsion. The balloon was left for 24 h and bleeding was controlled [7].

Apart from stopping bleeding from vaginal lacerations, the Bakri balloon has also been reported to prevent recurrent vaginal haematoma formation and to stop bleeding in hematocolpos. Gizzo et al. reported an obstetric patient with recurrent haematoma in the left ischiorectal fossa after vaginal delivery. He inserted a Bakri balloon in the vagina after a second attempt at surgical drainage of the haematoma. 3 L of saline was infused and the balloon was left for around 48 h [8]. Similarly, Yuksel B et al. described the use of Bakri balloon to stop the vaginal bleeding in a patient after LeFort colpocleisis performed for pelvic organ prolapse [9].

Apart from Bakri balloon, other types of balloon had been reported to be successful in PPH due to vaginal lacerations. Srivastava et al. described the use of a Rusch balloon in a patient with vaginal laceration after midcavity forceps delivery. The author did not state the amount of fluid used to inflate the Rush balloon, and no additional measures were used to keep the balloon in situ. The balloon was kept for 24 h [10]. Makin et al. used a condom balloon tamponade by fastening a condom on a foley catheter by a string and infusing 300 ml of water to control PPH in a patient having vaginal lacerations after normal vaginal delivery. Vaginal gauze was packed to prevent the balloon from slipping out. The condom balloon was kept for 48 h [11].

We have initially attempted to use a cervical ripening balloon in our patient. According to the manufacturer instructions, the maximum capacity of the vaginal balloon in the cervical ripening catheter was 80 ml, yet we were unable to control bleeding after infusing up to 100 ml saline. We therefore switched to use the Bakri balloon so that a larger volume could be infused to produce tamponade effect. Similar to the Tattersall approach [6], we applied a stitch to close the vulva to avoid balloon expulsion with good effect. Compared with vaginal gauze packing, we believe that the Bakri balloon is easier and quicker to be inserted and removed, should create less vaginal scarring and that the drainage tube in the system should allow continuous monitoring for any ongoing bleeding. In addition, we postulate that vaginal packing with balloon tamponade or a blood pressure cuff should produce a more uniform pressure tamponade effect similar as direct manual compression but probably superior to gauze packing, which requires more clinical skills and often fails to achieve the required haemostatic pressure. Compared to vaginal packing with a blood pressure cuff, the Bakri balloon tamponade is obviously less traumatic, simpler and quicker to apply.

**Fig. 1:**
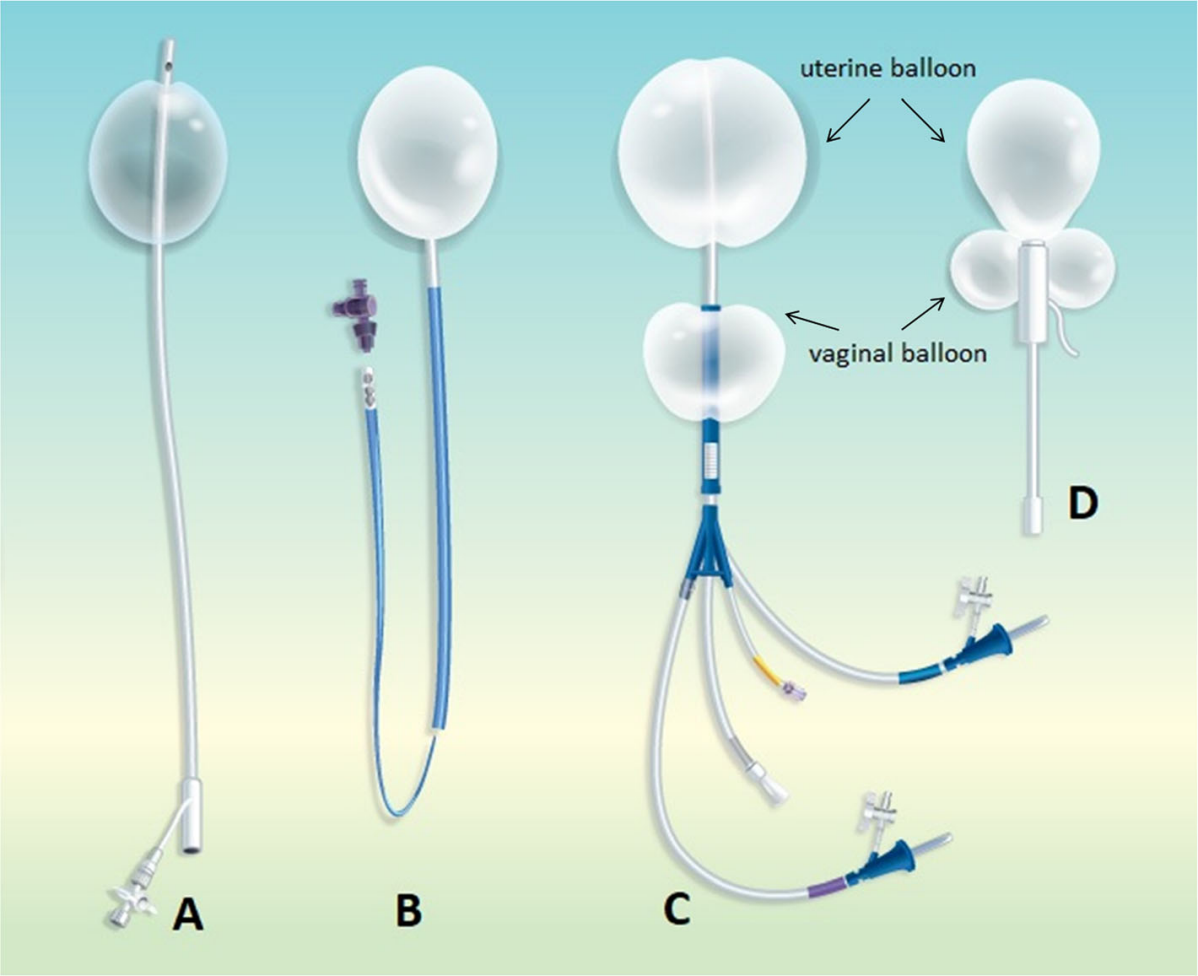
The different types of balloon tamponade specifically designed for use in postpartum hemorrhage. **a** Bakri balloon. **b** Vagistop. **c** Belfort-Dildy Complete Obstetrical Tamponade System (ebb’s balloon). **d** Zhukovsky obstetric balloon

In recent few years, several types of balloon tamponade systems have been manufactured which have specific vaginal balloons for use in PPH. The Vagistop (Ri.MOS., Medical Products, Mirandola, Italy) is a vaginal polymer balloon that is specifically designed for treating vaginal tears and haematoma. In a case series of 4 patients, the Vagistop successfully stopped bleeding in vaginal lacerations or in vaginal haematoma after normal vaginal deliveries and instrumental deliveries with 360–460 ml of air inflated into the balloon [12]. The author commented that the Vagistop was self-retaining in the vagina and no further interventions were needed to prevent balloon slipping from the vagina. The Belfort-Dildy Complete Obstetrical Tamponade System (ebb’s balloon) is a complete tamponade system with both uterine and vaginal balloon in a catheter [13]. The system was reported to be successfully used in a patient who had caesarean section for face presentation, but who suffered from PPH due to uterine atony and multiple vaginal tears [14]. The uterine and vaginal balloon were infused with 500 and 300 ml liquid respectively. This double balloon tamponade system should be ideal for patients with uterine and vaginal bleeding. However, in patients who already have a hysterectomy as in our patient, the uterine balloon becomes redundant and cannot be accommodated in the vagina. The Zhukovsky obstetric balloon consists of a uterine balloon and a vaginal balloon that can be inserted in combination or separately without the other. Therefore, the Zhukovsky system appears to offer flexibility for patients with both uterine and vaginal hemorrhage as well as in those with vaginal bleeding alone after peripartum hysterectomy. Barinov et al. showed that inflation of both the Zhukovsky uterine and vaginal balloons significantly reduced blood loss in caesarean section for placenta accreta patients [15]. For patients having bleeding from vaginal lacerations but with an intact uterus, double balloon devices such as the ebb tamponade system and the Zhukovsky obstetric balloon should offer additional advantage. The inflated uterine balloon in the uterine cavity can act as a fixator for the vaginal tamponade and no additional sutures in the vulva or vaginal gauze packing will be needed to prevent the vaginal balloon from slipping out. However, the Vagistop, the ebb tamponade system and the Zhukovsky obstetric balloon are currently not widely available in many parts of the world. The different types of balloon tamponade mentioned above are shown in Fig. 1 and their features are compared in Table 1.

**Table 1:** Comparison between different types of balloon tamponade

	Bakri	Vagistop	Ebb	Zhukovsky
Material	Silicon	Polymer	Polyurethane	Not mentioned by manufacturer
Presence of drainage tube	Yes	No	Yes	Yes
Had uterine balloon	✓	X	✓	✓
Had vaginal balloon	X	✓	✓ (The vaginal balloon cannot be separated from the uterine balloon)	✓ (The vaginal balloon can be separated from the uterine balloon and they can be inserted separately or can be inserted together)
Infusion medium	Fluid	Air	Fluid	Air/ Fluid
Maximum suggested infusion volume (ml)	500	500	750 (for uterine balloon) 300 (for vaginal balloon)	Not mentioned by manufacturer

As far as we are aware, there are no other case reports of stress incontinence as a complication after the use of vaginal balloon tamponade as in our patient. Prolonged vaginal balloon dilation was shown to cause stress incontinence, urodynamic changes and histological abnormalities in the urethra including reduced collagen content, fragmented elastic fibers, as well as sparsely arranged and shortened striated muscle fibers in the rat model [16, 17]. However, the degree of pelvic floor distension that would lead to such anatomical damage remains uncertain. Our patient had a single Bakri balloon infused with 300 ml saline in situ for 30 h. However, the case reported by Tattersall with two Bakri balloons with a total of 750 ml saline infused and placed in vagina for 30 h and the case reported by Gizzo with a Bakri balloon infused with 3 L of saline and placed for 48 h did not report stress incontinence afterwards. In addition, parity and vaginal delivery are known risk factors for stress incontinence. In the latest systematic review on prevalence of postpartum urinary incontinence found the prevalence of incontinence was 33% in all women while the mean prevalence of weekly and daily incontinence was 12 and 3% respectively. The prevalence was double in the vaginal group compared to caesarean section group [18]. Although stress incontinence is not rare after vaginal delivery, the daily severe stress incontinence in our patient requiring regular incontinence napkins was rare in postpartum women. We postulated that the vaginal balloon might cause prolonged compression in the pelvic floor with undue distension of the distal sphincters of the lower genitourinary tract, which may have stretched the nerve supply leading to stress incontinence. Indeed, Palacios et al. had reported vaginal balloon significantly increased the length of the motor branch of the sacral plexus, the dorsal nerve of the clitoris and vesical nerves. It also decreased the frequency and amplitude of firing of the dorsal nerve of the clitoris in a rat model [19]. The exact mechanism of how vaginal balloon can lead to stress incontinence would require further investigations. Our unit had investigated the intraluminal pressure of Bakri balloon placed in the uterine cavity and found its pressure was between 67 and 92 mmHg, but never exceeded the systematic blood pressure of the patient when less than the recommended maximal capacity of 500 ml saline was infused [20]. The actual intraluminal pressure in the Bakri balloon placed inside the vagina instead of the uterine cavity would require further investigations. Nevertheless, as vaginal balloon tamponade methods are increasingly used to control haemorrhage from severe vaginal lacerations or to prevent haematoma formation, we need to be aware of stress incontinence as a possible complication. Fortunately, as in the rat model study [16], the stress incontinence symptoms in our patient also gradually improved with time.

In conclusion, the use of Bakri balloon can help to control bleeding in severe vaginal lacerations that are unresponsive to traditional vaginal gauze packing and embolization. Further studies are needed to evaluate the risks of stress incontinence as a possible complication of vaginal balloon tamponade.

## Data Availability

Data are available from the corresponding author on reasonable request.

## Abbreviation

PPH: Postpartum hemorrhage
